# Investigating the acceptability of non-mesh, long-lasting insecticidal nets amongst nomadic communities in Garissa County, Kenya using a prospective, longitudinal study design and cross-sectional household surveys

**DOI:** 10.1186/s12936-015-0546-1

**Published:** 2015-02-05

**Authors:** Georgia R Gore-Langton, James Mungai, Nfornuh Alenwi, Abdulla Abagira, Owen M Bicknell, Rebecca Harrison, Farah A Hassan, Stephen Munga, Francis Njoroge, Elizabeth Juma, Richard Allan

**Affiliations:** The MENTOR Initiative, Crawley, UK; The MENTOR Initiative, Garissa, Kenya; Ministry of Health, Garissa, Kenya; The MENTOR Initiative, Maban, South Sudan; Kenya Medical Research Institute, Kisumu, Kenya; Ministry of Public Health, Nairobi, Kenya

**Keywords:** Malaria, LLINs, Non-mesh, Exophagy, Nomads, Acceptability, Utilization

## Abstract

**Background:**

North East Kenya is an area of semi-arid terrain, prone to malaria epidemics. The distribution of long-lasting insecticidal nets (LLINs) has long been a key malaria intervention, however, for nomadic populations who live and sleep outside, in harsh climates and areas with increasing reports of exophagic behaviour of mosquitoes, traditional LLINs are often inadequate. This study investigates the acceptability of non-mesh LLINs, specifically designed to suit nomadic, outdoor sleeping communities.

**Methods:**

In September 2011, 13,922 non-mesh LLINs were distributed to 8,511 nomadic households in Garissa County, North East Province, Kenya. A prospective, longitudinal study design was used to assess the acceptability of this novel type of LLIN. Cross-sectional household surveys, focus group discussions (FGDs), and key informant interviews (KIs) were used to collect data on attitudes and practices regarding the Dumuria nets.

**Results:**

A very high level of acceptability was reported with 95.3% of respondents stating they liked the nets. Of the factors reportedly determining net use the most frequently mentioned was “vulnerability”. Of those with concerns about the nets, the colour (white) was the most frequently reported.

**Conclusion:**

The tailoring of LLINs to specific communities and contexts leads to increased levels of acceptability. Large-scale, blanket net distribution campaigns, which are currently the standard practice, do not cater for the specific and nuanced needs of the differing communities they often serve. This non-mesh LLIN offers a highly effective and desirable malaria prevention option to a typically hard to reach and underserved nomadic population at increased risk of malaria infection.

## Background

Globally malaria caused 584,000 deaths in 2013 there were an estimated 198 million clinical cases [1]. Malaria control and treatment appears as one of eight Millennium Development Goals as “Goal 6: Combat HIV/AIDS, malaria and other diseases” [2]. In 2013 an estimated $2.7 billion was spent on malaria control, significantly below the estimated amount needed to achieve universal coverage of malaria interventions ($5.1 billion) [1]. Principle among these malaria interventions is the distribution of long-lasting insecticidal nets (LLINs) [3].

LLINs have been shown to be highly effective in many settings [4-8]; however, they are predicated upon use indoors. There have been increasing reports of exophagic vector behaviours playing a significant role in malaria transmission [9-11]. This is of particular relevance and concern to nomadic populations often found to sleep outdoors. Nomadic populations, defined here as groups of people with no fixed home who move according to the seasons and in search of water, food, and pasture, have been estimated at 50 – 100 million persons [12] with over 60% found in Africa, and making up approximately 19% of the population in Kenya [13,14]. Given this large population size, their pre-existing vulnerability due to reduced access to health care [12,14], and the increased threat exophagy poses to these outdoor sleeping populations, exploring the adequacies and limitations of LLINs in these contexts is a pressing issue.

In order for LLINs to be effective at preventing malaria, they must be correctly used every night. It is, therefore, necessary to identify factors that affect acceptability rates and to then use this information to create a more attractive and effective product. A review of the barriers to net use in malaria endemic areas, including Kenya, found that the most commonly cited reason for non-use of malaria nets was discomfort, primarily due to heat, accounting for 47.5% of reasons for not using a net. The second most frequently cited reason was perceived (low) mosquito density [15]. A study encompassing six locations across Western Kenya investigated the status of universal coverage of ITNs. One of the indicators was ITN usage- the proportion of the population that used ITNs the previous night. Results showed usage to range between 75 and 87%. Usage rates were roughly 10% lower than ownership rates [16].

**Figure 1:**
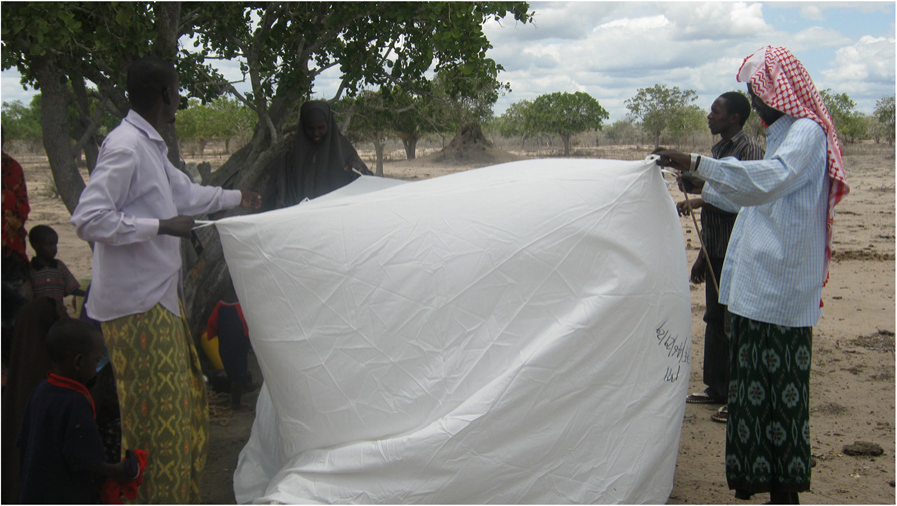
**The Dumuria LLIN.** A Dumuria net being displayed in the field.

PermaNet® Dumuria (hereafter referred to as a Dumuria net) is an LLIN produced by Vestergaard, Switzerland, intended for use both indoors and outdoors. This net is based on the PermaNet® 2.0 which is fully evaluated and recommended by the WHO Pesticide Evaluation Scheme (WHOPES) [17]; the only differences being unlike a typical 156 mesh LLIN this net is made of a non-mesh, opaque, bed sheet-like fabric (Figure 1) and added to the insecticide in the Dumuria net (and not found in the PermaNet® 2.0) are UV protectants designed to make the insecticide more resilient to sunlight exposure. This net has previously been distributed to the nomadic population of South Sudan where extremely high levels of acceptability were found when compared to standard LLINs (unpublished observations, P. Guillet).

A study assessing the durability of Dumuria nets at the study site and amongst the same population found it had good physical and chemical integrity after almost two years and high rates of utilization (Gore-Langton *et al*. personal communication). It is the purpose of this study to determine what the level of acceptability is for this novel, non-mesh LLIN designed specifically for use among outdoor sleeping populations and distributed among the study population of a nomadic community. Determining the level of acceptability and reasons for use or non-use of the Dumuria net is vital to the evaluation of this novel net and the benefits to malaria control that it may be able to offer typically underserved populations. This information is also hoped to guide the further development of this tool, and similar tools, so as to secure the highest possible levels of acceptability among the targeted population.

Presented here are the results from a 22-month longitudinal study of the acceptability of Dumuria LLINs amongst nomadic communities in Garissa County, North Eastern Kenya.

## Methods

### Study area and population

**Figure 2:**
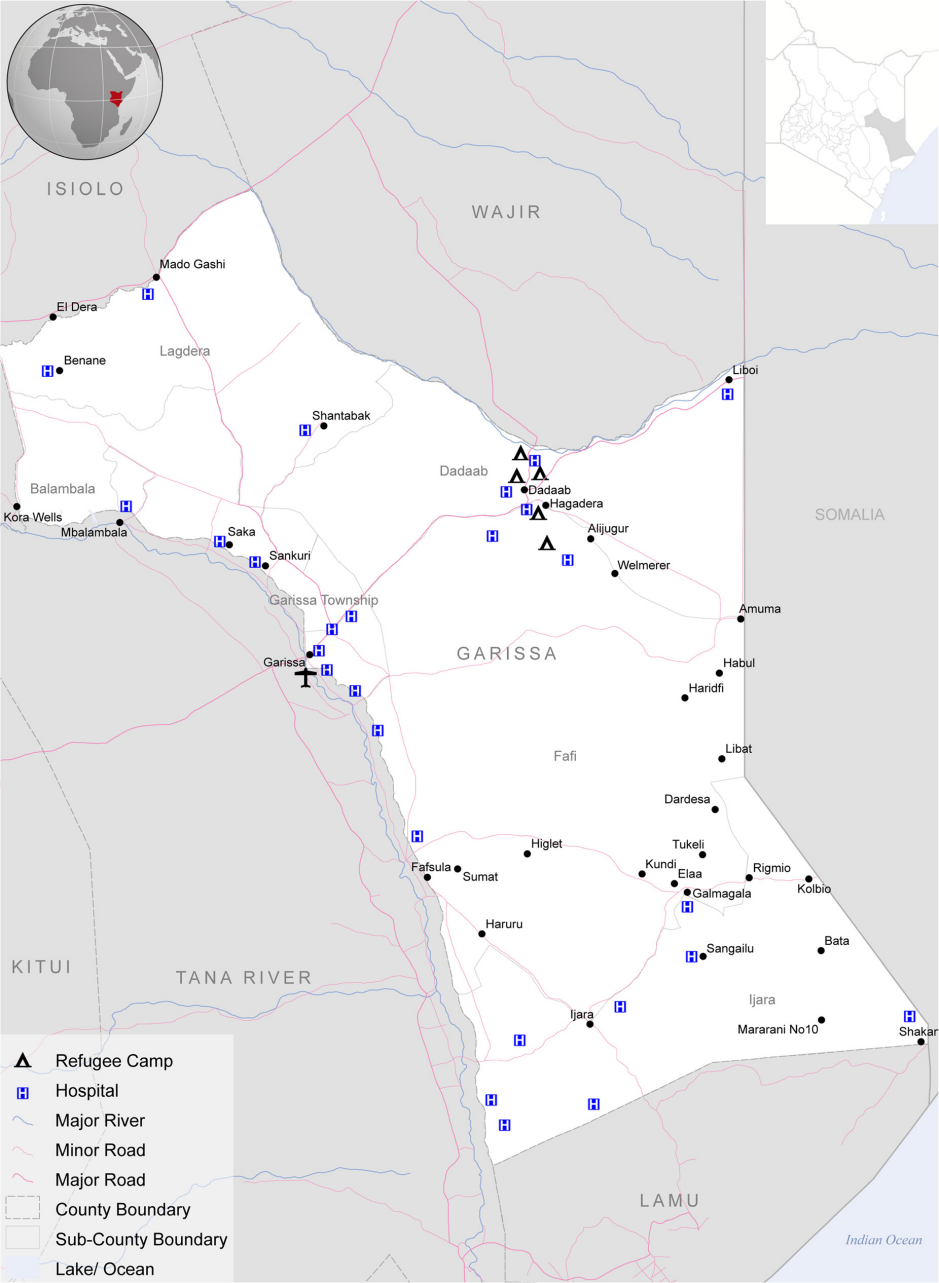
**Map of the study area.** A map of the study area of Greater Garissa, North Eastern Province, Kenya (from World Food Programme, 2006).

Garissa County is situated in North Eastern Province, Kenya (Figure 2); it is split into three administrative districts, Garissa, Lagdera, and Fafi, with a total of 11 divisions. The climate is semi-arid with a range in temperature from 21°C to 39°C in 2012 [18] and an annual average bimodal rainfall (rainy seasons from March – May and September – October) of 250 – 300 mm. The population according to the last national census in 2009 was 623,060 [19]. The population was estimated to have grown to 715, 312 in 2014 based on the Unicef prediction of a 2.8% annual growth rate [20].

Malaria transmission in Garissa is seasonal and epidemic prone [21]. The number of confirmed malaria cases per 1,000 population in this area is 0.1-1.0 [1]. The primary vectors in the region are *Anopheles coustani, Anopheles gambiae, Anopheles funestus,* and *Anopheles arabiensis* [1,22,23]. Each of these vectors has been shown to be both exophagic and exophilic across various study sites [24-28]. Both *Plasmodium falciparum* and *Plasmodium vivax* are present, with *P. falciparum* accounting for the majority of cases [29].

In September 2011, a total of 13,922 Dumuria nets were distributed to 8,511 nomadic households in Garissa county, providing an average coverage rate of 1.64 nets per household. This study area is one which at the time of the study was not within the national LLIN distribution plan as it is not an area of high malaria transmission, meaning that the study population had no previous, significant experience of LLINs [30]. All households received malaria prevention education to encourage correct net usage. Each net was assigned a unique identifier and added to a master census list along with a household code and global positioning satellites coordinates.

### Study design and sample size

From September 2011 to July 2013 a prospective longitudinal study was conducted with cross-sectional household surveys to assess Dumuria net utilization and acceptability at months 6, 12, 18, and 22. Households were randomly selected from all households that received the Dumuria nets using a two-stage cluster sampling method.

Sample size was calculated using the formula [*Np*(1 − *p*)]/[(*d*^2^/*Z*^2^_1 − *α*/2_ * (N − 1) + p * (1 − p)], with the conservative estimate of 60% retention and usage of Dumuria (p = 0.6) and a 95% confidence interval. Thirty clusters were randomly selected using the probability proportional to size method. Simple random sampling was used to select 10 households from each cluster to give a sample size of 300 for each round of surveys. Three hundred households were sampled at 6, 12, 18, and 22 months for a total of 1,200. Destructive sampling was followed, with the net being removed from the master list after being surveyed.

### Ethical approval

This study was planned with and approved by the Department of Malaria Control (DoMC), Ministry of Health in Garissa, and the Kenyan Medical Research Institute (KEMRI) and the Ethics Review Committee (ERC). All nomadic settlements involved in the survey were informed in advance of the net distribution and the surveys. Approval was obtained from local chiefs and traditional authorities and informed consent was obtained from the head of each household surveyed. The consent form was read to each participant in Somali by the interviewer. The respondent was then asked to sign/ thumb print the consent form if he/she accepted to participate in the study.

### Field procedures

The randomly selected households were visited and the questionnaires administered. Focus Group Discussions (FDGs) and Key informant Interviews (KIs) took place during month one and month 22 of the study. A total of 10 FGDs and 10 KIs were conducted by two teams of three researchers. Descriptive statistics and information on Dumuria net status, use, and handling were collected via questionnaires with a range of categorical responses allowing for “other” responses to be given. FGDs and KIs involved a range of open-ended questions to which participants could respond freely; responses were then grouped according to common content or theme. Survey questions were transcribed in Somali before being translated into English. All interviews were recorded by digital recorder and transcribed as soon as possible with the target of being done within 24 hours of the interview.

### Data handling and statistical analysis

Quantitative data were double entered into Epi-Info (WHO/CDC 2000) and analysed in SPSS (SPSS Inc. Released 2007. SPSS for Windows, Version 16.0. Chicago, SPSS Inc.) and STATA (StataCorp. 2013. *Stata Statistical Software: Release 13*. College Station, TX: StataCorp LP). Data were summarised using proportions and means. 95% confidence intervals were used where appropriate.

## Results

### Household characteristics

Overall, a total of 1,197 Dumuria nets were sampled from as many households, with a total of 7,365 inhabitants. The mean number of persons per household was 6.14 and 31.8% (95% CI: 30.7, 32.8) of the total population was under five years old. The median number of sleeping places per household was 2 (range: 2–22); what constituted a sleeping place differed between households. Sleeping places could be regular or temporary, they could be a bed, sand, bare floor, could be covered by bedding or not, and could be found either inside or outside. 54.3% (95% CI: 49.4, 59.7) of respondents had attended at least primary level education.

### Acceptability

From the aggregated data over the study time intervals and data collection methods 95.3% (95% CI: 93.5, 96.6) of respondents stated liking the Dumuria nets, 2.6% (95% CI: 1.6, 3.6) stated disliking the nets, 2.1% (95% CI: 1.3, 3.1) did not know, and there were 173 (14.5%) missing values. 58.1% (95% CI: 55.3, 60.9) of respondents reported liking the size of the nets, 35.6% (95% CI: 33.0, 38.4) reported liking the fabric, 33.6% (95% CI: 31.0, 36.3) reported liking the shape, and 14.2% (95% CI: 12.3, 16.3) of respondents reported liking the colour (white).

When asked which people were most likely to use the Dumuria nets 51% (95% CI: 48.2, 53.9) responded children under five, 25.9% (95% CI: 23.5, 28.5) responded children over five but under 15 years, 31.1% (95% CI: 28.5, 33.8) responded adults (over 15 years), 24.4% (95% CI: 22.0, 26.9) responded adults and children of all ages, and 10.1% (95% CI: 8.5, 12.0) responded mother and father only.

The most commonly mentioned factor that determined net use was ‘Vulnerability’ mentioned by 49.3% (95% CI: 46.5, 52.1) of respondents. For the purpose of this study, vulnerability referred to a person perceived to be at increased risk of malaria by the survey respondent; survey responses which stated “children <5 years” or “sick people” were included in this vulnerability criteria, pregnant women were not included as they were considered a separate factor for determining net use. 20% (95% CI: 17.8, 22.3) of respondents listed the season as a factor that determined net use. In response to the question ‘when do most people stop using the nets?’ 65.5% (95% CI: 62.81, 68.19) said during the dry season, 8.1% (95% CI: 6.55, 9.65) said at any time, 57.8% (95% CI: 55.0, 60.6) of all households stated that they stopped using the net when holes had appeared and 17% (95% CI: 14.87, 19.13) responded not applicable.

58.8% (CI 95%: 55.4, 62.2) of respondents reported having some sort of problem with the Dumuria net, although there were 388 (32.4%) missing values for this variable. The most frequently mentioned concern was the colour of the Dumuria net, followed by heat/ventilation, the least frequently reported concerns related to the fabric and washing of the net (Table 1).

**Table 1:** **Reported concerns regarding the Dumuria nets**

**Concern**	**Respondents reporting concern (%)**	**95% confidence interval**
Colour	47.1	43.1, 51.0
Heat/ventilation	26.6	23.3, 30.0
Shape	20.0	17.0, 23.3
Size	5.4	3.8, 7.4
Fabric and washing	0.5	0.2, 1.4

A breakdown of the different types of concerns reported by study participants in relation to use of the Dumuria nets, with the percentage of respondents reporting each specific concern and the 95% confidence intervals.

## Discussion

A highly durable LLIN containing an effective insecticide must also be of a design and style which make it accepted and liked by the population it is to protect. An LLIN which is disliked or unpopular risks not being used as it is designed to be and compromising the potential benefits to malaria control. It is, therefore, very encouraging that the overall acceptability of novel Dumuria nets, never before seen or used in this location or amongst this population, is very high with 95.3% of respondents stating they like the nets.

Despite the very high proportion of users liking the Dumuria nets, over half of respondents reported some sort of concern about the net and almost a third of all households did not respond to questions of concerns. Non-response could be evidence of courtesy bias with grateful recipients preferring to say nothing than anything negative; it could also be evidence of misunderstanding of the meaning or purpose of the questions. Whilst the numbers of missing values (n = 175, 14.5%) and “don’t know” responses (n = 21, 2.1%) are not negligible, if all these values are assumed to be negative responses then 81.3%, the majority, of all respondents liked the nets. Whilst courtesy bias should always be considered in longitudinal studies of this nature, results from another paper on the same study show high Dumuria net utilization rates of 98.4% at 22 months (Gore-Langton *et al*. personal communication). Since acceptability and utilization are not unrelated the high reported utilization rates provide further support for the acceptability results presented here.

Colour stood out as the most frequently mentioned concern making up almost half of all responses. The Dumuria nets are white and from conversations with the study participants this seemed to be a concern due to the ease with which they picked up dirt – as can be imagined in a semi-arid setting. It is conceivable that a white net requires more frequent washing and subsequent drying in the sun, which may affect the insecticidal activity of the Dumuria net. When asked about the preferred choice for the colour, most respondents preferred a darker colour (blue/black), which may not show the dirt so much. However, the second most frequently reported concern was heat/lack of ventilation and a darker coloured net may compound this issue, if only in the early evening and early morning when the sun is in the sky and adults and/or children may still be under the net. Whilst such high levels of acceptability over a significant period of time may make these concerns seem not all that pressing, they are important to consider as feedback on how to further improve and secure the highest possible acceptability rates of Dumuria nets.

The high levels of acceptability of Dumuria nets suggest a popular potential solution to current challenges in malaria prevention, key amongst which are increasing reports of exophagy and outdoor sleeping populations. Any potential shift from typically endophagic transmission is of particular concern to outdoor sleeping communities, such as the ones at the centre of this study. LLINs designed for indoor sleeping are often inadequate and/or unsuitable when used outside in some of the harshest climates in the world. Hence the need for novel LLINs designed to cope specifically with the effects of sunlight, and increased physical wear and tear, and which are liked and accepted by the people and communities they hope to protect. The Dumuria net appears a viable and promising answer to this malaria control problem.

## Conclusion

The nomadic populations subject of this study live in some of the harshest climates on earth, they sleep outside and live long distances from health centres, making traditional malaria control tools challenging to provide and often ineffective. The high levels of acceptance reported in this study provide support for the development and deployment of novel LLINs, tailored to the habits and needs of the recipient populations. This study shows that the designing and tailoring of Dumuria nets in order to best suit the communities and environments towards which they are targeted results in incredibly high levels of acceptability of the net. This study highlights the Dumuria net as a tool with great potential to protect long under-served and vulnerable communities living in areas of changing malaria risk, and in doing so challenges the one-net-fits all approach typically taken by mass distribution campaigns.

## Abbreviations

CDC: Centre for disease control
DoMC: Department of Malaria Control
ERC: Ethics Review Committee
FGD: Focus Group Discussion
KEMRI: Kenyan Medical Research Institute
KI: Key informant Interview
LLIN: Long-lasting insecticidal net
WHO: World Health Organization

## References

[1] WHO (2014). World Malaria Report 2014.

[2] United Nations Millennium Development Goals [http://www.un.org/millenniumgoals/]

[3] WHO. Recommendations for Achieving Universal Coverage with Long-lasting Insecticidal Nets in Malaria Control. World Health Organization. 2013.

[4] Lengeler C. Insecticide-Treated Bed Nets and Curtains for Preventing Malaria. Cochrane Database Syst Rev. 2004;CD000363. 10.1002/14651858.CD000363.pub2 15106149

[5] Lim SS, Fullman N, Stokes A, Ravishankar N, Masiye F, Murray CJL (2011). Net benefits: a multicountry analysis of observational data examining associations between insecticide-treated mosquito nets and health outcomes. PLoS Med.

[6] Curtis CF, Mnzava AE (2000). Comparison of house spraying and insecticide-treated nets for malaria control. Bull World Health Organ.

[7] Eisele TP, Larsen DA, Walker N, Cibulskis RE, Yukich JO, Zikusooka CM (2012). Estimates of child deaths prevented from malaria prevention scale-up in Africa 2001–2010. Malar J.

[8] Okumu FO, Mbeyela E, Lingamba G, Moore J, Ntamatungiro AJ, Kavishe DR (2013). Comparative field evaluation of combinations of long-lasting insecticide treated nets and indoor residual spraying, relative to either method alone, for malaria prevention in an area where the main vector is Anopheles arabiensis. Parasit Vectors.

[9] Kitau J, Oxborough RM, Tungu PK, Matowo J, Malima RC, Magesa SM (2012). Species shifts in the *Anopheles gambiae* complex: do LLINs successfully control *Anopheles arabiensis*?. PLoS One.

[10] Tirados I, Costantini C, Gibson G, Torr SJ (2006). Blood-feeding behaviour of the malarial mosquito *Anopheles arabiensis*: implications for vector control. Med Vet Entomol.

[11] Coosemans M, Lies D. Residual Transmission of Malaria: An Old Issue for New Approaches. In Anopheles Mosquitoes - New Insights into Malaria Vectors. Edited by Manguin S. InTech; 2013.

[12] Omar MA (1992). Health care for nomads too, please. World Health Forum.

[13] Kenya: RAPID BASELINE ASSESSMENT WITH EXCLUSIVE FOCUS ON PASTORALIST DROP-OUTS (GARISSA MUNICIPALITY) - Kenya | ReliefWeb. Available at: http://reliefweb.int/report/kenya/kenya-rapid-baseline-assessment-exclusive-focus-pastoralist-drop-outs-garissa. Accessed March 4, 2014.

[14] Sheik-Mohamed A, Velema JP (1999). Where health care has no access: the nomadic populations of sub-Saharan Africa. Trop Med Int Health.

[15] Pulford J, Hetzel MW, Bryant M, Siba PM, Mueller I (2011). Reported reasons for not using a mosquito net when one is available: a review of the published literature. Malar J.

[16] Zhou G, Li JS, Ototo EN, Atieli HE, Githeko AK, Yan G (2014). Evaluation of universal coverage of insecticide-treated nets in western Kenya: field surveys. Malar J.

[17] WHO. Report of the Twelfth WHOPES Working Group Meeting. 2008, World Health Organization, WHO/HTM/NTD/WHOPES/2009.1.

[18] Historical Weather For 2012 in Garissa, Kenya - WeatherSpark [http://weatherspark.com/history/29260/2012/Garissa-North-Eastern-Kenya]

[19] 2009 Kenya Population and Housing Census Highlights. 2009. [http://www.scribd.com/doc/36672705/Kenya-Census-2009#scribd]

[20] Unicef. Maternal and Newborn Health. New York. 2008. [http://www.unicef.org/sowc09/docs/SOWC09-FullReport-EN.pdf]

[21] Division of Malaria Control [Ministry of Public Health and Sanitation], Kenya National Bureau of Statistics, and ICF Macro. 2011. 2010 Kenya Malaria Indicator Survey. Nairobi, Kenya: DOMC, KNBS and ICF Macro. [http://dhsprogram.com/pubs/pdf/MIS7/MIS7.pdf]

[22] Lutomiah J, Bast J, Clark J, Richardson J, Yalwala S, Oullo D (2013). Abundance, diversity, and distribution of mosquito vectors in selected ecological regions of Kenya: public health implications. J Vector Ecol.

[23] Sang R, Kioko E, Lutomiah J, Warigia M, Ochieng C, O’Guinn M (2010). Rift Valley fever virus epidemic in Kenya, 2006/2007: the entomologic investigations. Am J Trop Med Hyg.

[24] Brooke BD, Kloke G, Hunt RH, Koekemoer LL, Temu EA, Taylor ME (2001). Bioassay and biochemical analyses of insecticide resistance in southern African *Anopheles funestus* (Diptera: Culicidae). Bull Entomol Res.

[25] Moiroux N, Gomez MB, Pennetier C, Elanga E, Djènontin A, Chandre F (2012). Changes in *Anopheles funestus* biting behavior following universal coverage of long-lasting insecticidal nets in Benin. J Infect Dis.

[26] Sougoufara S, Diédhiou SM, Doucouré S, Diagne N, Sembène PM, Harry M (2014). Biting by *Anopheles funestus* in broad daylight after use of long-lasting insecticidal nets: a new challenge to malaria elimination. Malar J.

[27] Kabbale FG, Akol AM, Kaddu JB, Onapa AW (2013). Biting patterns and seasonality of *Anopheles gambiae sensu lato* and *Anopheles funestus* mosquitoes in Kamuli District, Uganda. Parasit Vectors.

[28] Mwangangi JM, Muturi EJ, Muriu SM, Nzovu J, Midega JT, Mbogo C (2013). The role of *Anopheles arabiensis* and *Anopheles coustani* in indoor and outdoor malaria transmission in Taveta District, Kenya. Parasit Vectors.

[29] National Malaria Strategy 2009-2017, July 2009, Division of Malaria Control, Ministry of Public Health and Sanitation. [http://www.c-hubonline.org/sites/default/files/resources/main/Kenya_National_Malaria_Strategy_2009-2017.pdf]

[30] Malaria Operational Plan, KENYA, FY 2011. 2011.

